# High Level Resistance against Rhizomania Disease by Simultaneously Integrating Two Distinct Defense Mechanisms

**DOI:** 10.1371/journal.pone.0051414

**Published:** 2012-12-20

**Authors:** Ourania I. Pavli, Anastasia P. Tampakaki, George N. Skaracis

**Affiliations:** 1 Department of Crop Sciences, Agricultural University of Athens, Athens, Greece; 2 Department of Agricultural Biotechnology, Agricultural University of Athens, Athens, Greece; Cinvestav, Mexico

## Abstract

With the aim of achieving durable resistance against rhizomania disease of sugar beet, the employment of different sources of resistance to *Beet necrotic yellow vein virus* was pursued. To this purpose, *Nicotiana benthamiana* transgenic plants that simultaneously produce dsRNA originating from a conserved region of the BNYVV replicase gene and the HrpZ*_Psph_* protein in a secreted form (SP/HrpZ*_Psph_*) were produced. The integration and expression of both transgenes as well as proper production of the harpin protein were verified in all primary transformants and selfed progeny (T1, T2). Transgenic resistance was assessed by BNYVV-challenge inoculation on T2 progeny by scoring disease symptoms and DAS-ELISA at 20 and 30 dpi. Transgenic lines possessing single transformation events for both transgenes as well as wild type plants were included in inoculation experiments. Transgenic plants were highly resistant to virus infection, whereas in some cases immunity was achieved. In all cases, the resistant phenotype of transgenic plants carrying both transgenes was superior in comparison with the ones carrying a single transgene. Collectively, our findings demonstrate, for a first time, that the combination of two entirely different resistance mechanisms provide high level resistance or even immunity against the virus. Such a novel approach is anticipated to prevent a rapid virus adaptation that could potentially lead to the emergence of isolates with resistance breaking properties.

## Introduction

Sugar beet (*Beta vulgaris* L. ssp. *vulgaris*) is one of the most important industrial crop species, occupying globally a cultivated area of approximately 8.1 million hectares spread over 41 countries [1]. Worldwide, the economic viability of sugar beet cultivation is largely depended on the successful protection against pathogens, of which Rhizomania, a viral root disease caused by *Beet necrotic yellow vein virus* (BNYVV), exerts a very high impact. The disease causes severe economic losses as a consequence of a dramatic reduction in root yield, sugar content and purity, especially when infections occur early in the growing season [2]. In most of the rhizomania infested areas, partially resistant sugar beet varieties have replaced susceptible cultivars since they could lose up to 80–90% of their potential sugar yield [3].

BNYVV is a rod-shaped virus with a multipartite plus ssRNA genome, consisting of four genomic messenger-sense RNAs, with some isolates harbouring a fifth RNA segment [4]–[6]. RNAs 1 and 2 carry all necessary information for housekeeping functions, whereas the natural infection process requires the host-specific function of additional proteins, directly involved in pathogenesis and vector transmission, encoded by the small RNA species [7]. On the basis of its molecular characteristics, BNYVV has been classified in three major pathotypes, referred to as A, B, and P [8], [9]. BNYVV isolates containing a fifth RNA species are generally considered as more aggressive than those containing RNAs 1–4 [4], [10], [11]. A is the most widespread type, found in most EU countries, USA, China and Japan, whereas the other two types present a more limited spread [12], [13].

Rhizomania incidence and severity cannot be considerably reduced by preventive cultural practices such as rotation, avoidance of excessive soil moisture and early plantings. Consequently, the only substantial means to ensure a viable crop production in rhizomania incidence areas is the use of varieties specifically bred as resistant to the disease [14]. In this respect, coping with rhizomania to date has mainly been based on cultivars endowed with the *Rz1* gene (“Holly” source), a dominant gene conferring sufficiently high levels of protection against BNYVV [15]–[17]. In terms of generating high level disease resistance, the potential of various genetic engineering approaches has been further explored. Although successful at varying levels, all such methodologies have to date exploited the introgression of a single transgene with the perspective of engineering resistance based on either the expression of gene products of pathogen [18] or non-pathogen origin [19], [20] or the RNA-silencing signaling route [21]–[23].

However, recent changes in field and molecular BNYVV epidemiology, as manifested by the recent emergence of resistance-breaking mutants for the *Rz1*-mediated resistance, resulting from high selective pressure to overcome this gene [1], [13], [24]–[31], probably reflect an ensuing new endemic disease development. Such an evolution would require major adjustments in mainstream breeding programs if they were to keep providing a durable crop protection through the use of appropriate cultivars. Due to the limited availability of useful natural genetic sources of resistance against the prevailing virus strains [32] as well as the recent emergence of novel resistance breaking strains, all relevant breeding activities become of paramount importance.

In this study we explored the possibility to integrate two entirely different functional defense mechanisms in order to generate transgenic resistance against rhizomania disease of sugar beet. Towards this direction, we produced transgenic *N. benthamiana* plants simultaneously expressing two distinct transgenes: the *hrpZ_Psph_* from *P. syringae.* pv. *phaseolicola*, which has been previously proved as a valuable tool for achieving rhizomania resistance, and a conserved region of the BNYVV replicase gene arranged as inverted repeat, an arrangement known to act as a strong silencing inducer.

## Materials and Methods

### Plasmids and bacterial strains

*Agrobacterium tumefaciens* strain C58C1 harboring the binary plant expression vector construct pBin.Hyg.Tx-SP/*hrpZ_Psph_* [33] and *A. tumefaciens* strain GV3101 carrying the intron-hairpin construct GW-IR3 [23] were used to transform *N. benthamiana* plants. The former plasmid contains the *hrpZ* gene from *P. syringae.* pv. *phaseolicola* NPS3121 (approx. 1 kb) fused in-frame with the signal peptide from the tobacco pathogenesis-related protein, whereas the latter carries a fragment (459 bp) of the BNYVV RNA 1-encoded replicase gene, which upon transcription is processed as dsRNA.

Bacterial cells were grown at 28°C in liquid LB selection medium containing: rifampicine (50 µg ml^−1^), carbenicillin (100 µg ml^−1^) and kanamycin (50 µg ml^−1^) for strain C58C1; rifampicine (20 µg ml^−1^), gentamycin (25 µg ml^−1^) and spectinomycin (50 µg ml^−1^) for GV3101, for 2 days or until OD_600_ = 0.6–1 was reached. Cultures were centrifuged and then resuspended to a final concentration of 10^7^ cfu/ml. Cell suspensions were mixed in equal volumes and the resulting mixture was used as inoculum for plant transformation.

For the generation of transgenics carrying only one transgene, used as controls, cell suspensions were separately used for plant transformation.

### Plant transformation and evaluation of transgenics

For plant transformation, leaf discs from healthy *N. benthamiana* plants (5–6 weeks-old) were used as explants, according to the protocol described by Horsch et al. [34]. Following selection of transformants with respect to antibiotic resistance to hygromycin (30 µg ml^−1^) and kanamycin (150 µg ml^−1^) for the transforming plasmids pBin.Hyg.Tx-SP/*hrpZ_Psph_* and GW-IR3 respectively, regenerated shoots were rooted and transferred to soil.

Transgene integration and absence of Ti plasmid-originated sequences was examined by a multiplex PCR assay, using specific primers to amplify the 995 bp, 459 bp and 590 bp fragments of *hrpZ_Psph_* [20], the BNYVV-derived transgene [23] and *virG* of *A. tumefaciens* [20]. Transgene expression was verified by RT-PCR using abovementioned primers. Plants that were PCR-positive for transgene integration and expression were subsequently examined for the accumulation of SP/HrpZ*_Psph_* by immunoblot blot analysis as previously described [20]. Plants that met all above requirements were selfed and thereof progeny seeds (T1, T2) were selected against hygromycin and kanamycin. Resistant plants were grown *in vitro* for shoot and root formation and were subsequently transferred to soil. Representative T2 transgenic plants were further assessed on the basis of BNYVV-derived transgene expression and SP/HrpZ*_Psph_* protein accumulation.

### Virus inoculations

For foliar rub-inoculations of *N. benthamiana* plants, heavily BNYVV-infected sugar beet plants were exploited as virus source. In order to evaluate resistance under high infectivity pressure, a previously characterized as highly infectious virus isolate, due to the presence of specific aa at key positions of the BNYVV p25 protein, was used [31].

Transgenic lines simultaneously expressing SP/HrpZ*_Psph_* and BNYVV-derived dsRNA were assessed for resistance to the BNYVV. In addition, transgenic lines carrying only one transgene, either SP/*hrpZ_Psph_* [20] or BNYVV-dsRNA (this study), were also included in the experiments as comparative controls. In total, 30 transgenic *N. benthamiana* T0 lines were evaluated for BNYVV resistance: i) ten doubly transformed plants expressing SP/HrpZ*_Psph_* and BNYVV-derived dsRNA, ii) ten lines expressing SP/HrpZ*_Psph_* and iii) ten lines expressing the virus-derived transgene. Twelve T2 plants from each transgenic line were challenge-inoculated with BNYVV and were evaluated for resistance in two consecutive resistance assays.

Plants at the stage of 4–6 leaves were mechanically inoculated by first dusting at least 3 leaves with carborundum, in order to facilitate virus entrance, and then applying a virus-infected leaf extract. The inoculum source was obtained as described in Pavli et al. [23]. Inocula were tested on non-transgenic control *N. benthamiana* plants to confer infectivity. Tissue from non-transgenic uninoculated plants was accordingly processed and used as experimental negative control.

### Evaluation of virus resistance

Assessment of virus resistance in transgenic *N. benthamiana* was performed on the basis of macroscopically scoring disease symptoms and measuring virus titers. Plants were regularly monitored and symptoms were scored during the 30 day period post inoculation (dpi). The relative amount of virus in inoculated plants was estimated by DAS-ELISA (Adgen Phytodiagnostics) at 20 and 30 dpi, according to the supplier's instructions. Replicate leaf tissue samples were homogenized in 1∶3 extraction buffer. Samples scoring absorbance values (405 nm) at least three times the respective measurements of the negative controls were considered as positive.

## Results and Discussion

### Generation of transgenic *N. benthamiana* plants simultaneously expressing *SP/hrpZ_Psph_* and BNYVV replicase-derived transgene

The simultaneous expression of two distinct transgenes has been pursued for a first time as a means to develop high level and stable resistance against this most devastating disease of the sugar beet crop. In this context, a conserved region originating from the BNYVV replicase gene, arranged as inverted repeat, as well as the harpin SP/HrpZ*_Psph_* from *P. syringae* pv. *phaseolicola* have been simultaneously produced in transgenic *N. benthamiana* plants.

*A. tumefaciens*-mediated transformation of *N. benthamiana* plants with two distinct plasmids, pBin.Hyg.Tx-SP*/hrpZ_Psph_* and GW-IR3, was carried out by a standard leaf disc method. Sixteen (16) independent transgenic lines (T0) expressing SP/*hrpZ_Psph_* along with BNYVV-derived dsRNA were developed and ten of them were randomly selected and selfed to produce T1 and T2 lines.

Primary double transgenics (T0) and selfed progeny (T1, T2) were phenotypically normal at a macroscopic level, presenting however an increased vigor in comparison to the GW-IR3-transformed lines or to the non-transgenic plants of the same age. This phenotype has been previously ascribed to the expression of SP*/hrpZ_Psph_* [20]. In addition, such enhanced growth properties have been reported to occur both upon external application of purified harpins [35]–[38] and transgene expression [39]–[41].

**Figure 1:**
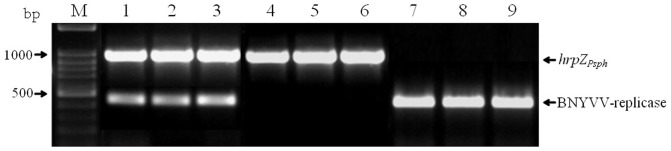
Amplification products obtained by multiplex PCR on total genomic DNA from *A. tumefaciens*-transformed *N. benthamiana* plants. Lane M: Marker in bp (Gene Ruler Ladder mix, Fermentas). Lanes 1, 2, 3: Representative T2 double transgenic plants, carrying the 995 bp fragment of *hrpZ_Psph_* and the 459 bp fragment of the BNYVV-replicase gene. Lanes 4, 5, 6: Representative T2 transgenic plants, carrying the 995 bp fragment of *hrpZ_Psph_*. Lanes 7, 8, 9: Representative T2 transgenic plants, carrying the 459 bp fragment of the BNYVV-replicase gene. The 590 bp amplicon corresponding to *virG* of *A. tumefaciens* could not be obtained, thus verifying the absence of Ti plasmid sequences in antibiotic resistant *N. benthamiana* transformants.

**Figure 2:**
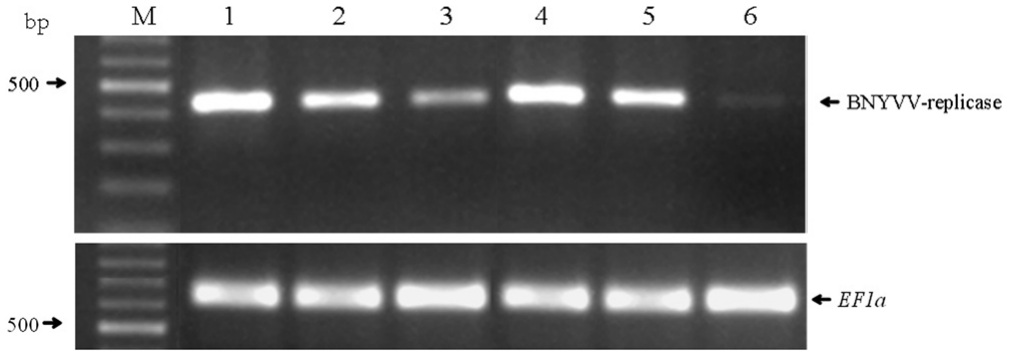
RT-PCR amplification products obtained from transgenic *N. benthamiana* plants expressing the BNYVV replicase-derived transgene. Lane M: Marker in bp (Gene Ruler Ladder mix, Fermentas). Lanes 1, 2, 3: Representative T2 double transgenic plants, expressing the *hrpZ_Psph_* and the BNYVV replicase-derived transgenes, presenting high, moderate and low resistance levels, respectively. Lanes 4, 5, 6: Representative T2 GW-IR3-transformed plants presenting high, moderate and low resistance levels, respectively. The *EF1a* gene transcript is used as loading control for equal amounts of RNA.

Proper transgene integration and absence of *A. tumefaciens* was verified by a multiplex PCR assay, targeting regions of *hrpZ_Psph_*, BNYVV-replicase gene and *virG* of the disarmed Ti plasmid, in all primary transgenics (T0) and selfed progeny (T2) tested. Figure 1 shows the presence of *hrpZ* and BNYVV-replicase region amplicons in representative T2 double transgenic plants as well the presence of the corresponding amplicons in transgenic plants carrying single transgenes. At the same time, the absence of *virG* amplicons confirms the transgenic nature of plants grown under antibiotic selection (data not shown). Transgene expression was verified, though at varying levels, by means of a RT-PCR assay, in more than 90% of the samples examined (Figure 2). More specifically, transgene expression was detected in 57 out of 60 T2 double transgenics and of these, 44 plants showed high expression levels whereas, 11 and 2 plants showed moderate and low transgene expression levels, respectively. In case of single transgenic plants, 54 were found positive for the BNYVV-derived transgene expression, and of these 27, 16 and 11 plants showed high, moderate and low expression levels, respectively.

**Figure 3:**
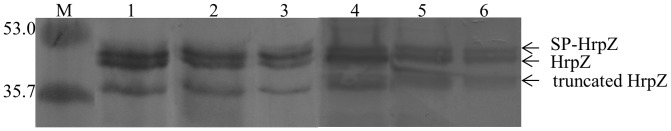
Western blot analysis of SP/HrpZ*_Psph_* in protein extracts from leaves of double and single transgenic *N. benthamiana* plants. Lane M: Marker in kDa (Broad range pre-stained SDS marker, Biorad). Lanes 1, 2, 3: Representative T2 double transgenic plants transformed with the plasmids pBin.Hyg.Tx-SP*/hrpZ_Psph_* and GW-IR3 showing high, moderate and low resistance levels, respectively. Lanes 4, 5, 6: Representative T2 plants transformed with the plasmid pBin.Hyg.Tx-SP*/hrpZ_Psph_* showing high, moderate and low resistance levels, respectively. The lower band represents the truncated form of the harpin (approx. 2 kDa smaller than the full-length HrpZ*_Psph_*). Equal amounts of total protein (30 ug) were loaded.

Immunoblot analysis of all primary transgenics (T0) and T2 transgenic lines tested indicated the production of three immunoreactive bands corresponding to SP/HrpZ, HrpZ and a truncated form of the protein (Figure 3). This result demonstrated that the protein was indeed produced and the signal peptide was properly processed and it is also consistent with a previous study [20].

### *N. benthamiana* plants expressing SP/HrpZ_Psph_ and BNYVV-derived dsRNA show high-level resistance to BNYVV inoculation

The ability of the two distinct transgenes, SP/*hrpZ_Psph_* and BNYVV-derived dsRNA, in conferring resistance to rhizomania disease was evaluated by BNYVV-challenge inoculation of double transgenic plants (T2 progeny) of *N. benthamiana*, which represents a model crop that can be readily exploited for the purpose of assessing rhizomania resistance as previously described [19]–[21]. Resistance was assessed by visual symptom observation as well as by measurement of virus titers using ELISA at 20 and 30 dpi.

In order to examine resistance of double transformed lines in comparison to lines carrying single transgenes, thirty transgenic *N. benthamiana* lines T0 were evaluated for BNYVV resistance: i) ten double transformed plants expressing both SP/HrpZ*_Psph_* and BNYVV-derived dsRNA, ii) ten lines expressing SP/HrpZ*_Psph_* and iii) ten lines expressing the BNYVV-derived transgene. Six progeny plants (T2), for each of the ten transgenic lines (T0) per group, were evaluated for virus infection. Challenge inoculation experiments were repeated twice by choosing different T2 plants in each experiment. In total, twelve T2 plants for each of the ten T0 lines (in total 120 T2 plants per group) were evaluated for resistance.

In non-transgenic plants, symptoms of leaf curling and sporadic chlorosis appeared at 10–14 dpi, whereas all of them exhibited systemic disease symptoms i.e. severe mosaic, occasional leaf distortion and general stunting, at 20–22 dpi. In addition, ELISA readings were in agreement with the abovementioned visual assessments (Table 1). Instead, transgenic plants expressing the *SP/hrpZ_Psph_* were highly resistant to BNYVV in terms of both symptom development and virus accumulation. Such results are in agreement with previous observations [20] and further substantiate the conclusion that the endogenous expression of SP*/hrpZ_Psph_* confers enhanced resistance against rhizomania. It is worth noting however, that the SP/HrpZ*_Psph_*-based resistance reported in this study, although quantitatively similar to the one earlier demonstrated [20], was assessed in T2 progeny, as opposed to our previous experiments conducted on T1 transgenic plants. Transgenic plants expressing a fragment of the virus replicase gene, which is transcribed in dsRNA, were challenge-inoculated and assessed for resistance to the BNYVV. Four out of the ten transgenic lines (T0) tested showed high level resistance with respect to symptom development and virus content, pointing to a mechanism analogous to that resulting in resistant sugar beet hairy roots transgenically expressing the BNYVV replicase-derived dsRNA [23]. In contrast, the T2 progeny of the remaining six lines (T0) segregated in varying levels of virus infection ranging from complete resistance to susceptibility (Table 1). It is worth mentioning, that highly resistant lines as well as resistant plants originating from segregating lines were characterized by high transgene expression levels whereas, susceptible plants showed low transgene expression levels. Such findings indicate that line performance is correlated to the corresponding level of the BNYVV replicase transgene expression.

**Table 1:** Symptom severity and virus titer of transgenic *N. benthamiana* T2 plants expressing SP/HrpZ*_Psph_* and BNYVV-derived dsRNA in comparison to T2 plants expressing either SP/HrpZ*_Psph_* or BNYVV-derived dsRNA, 30 days after challenge with BNYVV.

Plant type	T2 plants tested	T0 origin	1Symptom development			2Virus content		
			Score	Number of plants		Score	Number of plants	
		8	−	96^3^	80.0^4^	−	96	80.0
SP/HrpZ*_Psph_*			−	6	5.0	−	4	3.33
&	120					+	2	1.67
BNYVV-dsRNA		2	+	16	13.33	+	12	10.0
						++	4	3.33
			++	2	1.67	++	2	1.67
		7	−	84	70.0	−	78	65.0
						+	6	5.0
			−	10	8.33	+	10	8.33
SP/HrpZ*_Psph_*	120		+	18	15.0	+	16	13.33
		3				++	2	1.67
			++	8	6.67	++	5	4.17
						+++	3	2.5
		4	−	48	40.0	−	40	33.33
						+	8	6.67
			−	9	7.5	−	6	5.0
BNYVV-dsRNA	120					+	3	2.5
		6	+	35	29.17	+	27	22.5
						++	8	6.67
			++	28	23.33	++	23	19.16
						+++	5	4.17
**Non-transgenics**	30		+++	30	100^3^	+++	30	100

1 − absence of symptoms, + leaf curling, ++ faint mosaic, mild stunting, +++ severe mosaic, leaf distortion, general stunting.

2 −− value indicative of virus absence, + close to the positive threshold (three times the negative control), ++ half reading of positive control (non-transgenic), +++ equal to the positive control.

3 numbers denote number of plants in corresponding categories.

4 numbers denote percent of plants in corresponding categories.

**Figure 4:**
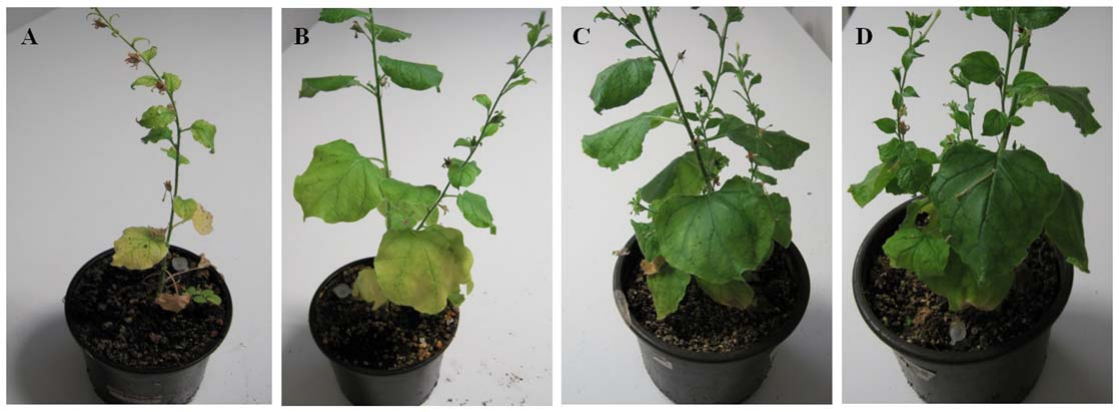
Symptoms of *Beet necrotic yellow vein virus* in *N. benthamiana* at 30 days post inoculation. A) Non-transgenic plant. B) T2 transgenic plant expressing fragment of the BNYVV-replicase gene. C) T2 transgenic plant expressing the *hrpZ_Psph_* transgene. D) T2 double transgenic plant expressing the *hrpZ_Psph_* and a fragment of the BNYVV-replicase gene. The transgenic plant expressing the *hrpZ_Psph_* as well as the double transgenic plant, expressing both the *hrpZ_Psph_* and the BNYVV-replicase transgene, are symptomless. In contrast, the BNYVV dsRNA-expressing plant and the non-transgenic control plant exhibit symptoms of general stunting and/or leaf curling.

Upon challenge with BNYVV, all T2 transgenic plants expressing both the SP*/hrpZ_Psph_* and the virus-derived transgene were characterized as highly resistant or even immune to infection. On the basis of symptom expression, the majority of T2 plants (85.0%) remained completely symptomless throughout the period of observation, whereas the remaining plants (15.0%) showed either leaf curling (13.33%) or mild stunting (1.67%), which were though delayed by approximately 15 days compared to the non-transgenic ones (Table 1) (Figure 4). More specifically, all the T2 plants deriving from eight of the ten Τ0 lines (96 plants) were entirely symptomless and virus-free as evidenced by visual scoring and ELISA assay, respectively (80.0%). The T2 progeny of the remaining two T0 lines were also partially resistant with a portion of them (6 plants) remaining symptomless (25.0%), while the rest 18 plants (75.0%) manifested mild to moderate disease symptoms and relatively low virus titers. At 20 dpi, the great majority of transgenic plants (91.4%) were negative to infection according to ELISA values (data not shown). At 30 dpi, 83.33% of these plants remained completely virus-free while 16.67% presented very low or marginally positive values (Table 1). These findings highlight the effectiveness of the simultaneous expression of the SP*/hrpZ_Psph_* and the replicase transgenes in conferring high level resistance or even immunity against BNYVV. At the same time, it has been demonstrated that such resistance is superior to the SP*/hrpZ_Psph_*-based resistance [20] or to the RNA silencing-mediated resistance described in this study (data presented in Table 1). Collectively, these data support the conclusion that although the single expression of abovementioned transgenes resulted in enhanced resistance against the disease, their co-expression in transgenic plants led to superior performance, presumably due to additive transgene effects.

Previously, it has been shown that the BNYVV-resistant phenotype is positively correlated with harpin accumulation to the plant cell exterior and it was further speculated that the observed resistance may be attributed to a primed state of plants expressing the SP/*hrpZ_Psph_* [20]. Furthermore, an intriguing observation was the development of a localized necrosis only in the SP/*hrpZ_Psph_*-expressing leaves in the virus inoculation area. Authors suggested that this necrosis might be attributed to an augmentation or synergistic effects of defense responses elicited by the extracellularly targeted harpin and virus infection. It is interesting that double transformants did not develop an analogous necrosis in the virus inoculation area. It is likely that the primed state of SP/HrpZ*_Psph_*-expressing plants mounts a first basal level of defense which may be subsequently enhanced by the RNA-silencing signaling route. In this context, it is tempting to speculate that such network interference or combined action of the two defense mechanisms may result in high disease resistance and absence of visible cell death.

A thorough investigation of the transcriptomic profile of defense-related genes in plants expressing SP/HrpZ*_Psph_* and BNYVV-derived dsRNA is anticipated to shed light in the resistance observed in this study. Furthermore, it is important to investigate the occurrence of siRNAs prior and at different time intervals post inoculation in order to determine the timing of the RNA silencing activation.

Conclusively, our findings highlight the potential of integrating two genetically distinct defense mechanisms as a novel tool to achieve high level and stable resistance against rhizomania disease of sugar beet. Such “co-targeting” approaches may be extended to a large number of viruses and/or other biotic agents and to a wide variety of cultivated crop species.

## References

[1] Rush CM, Liu H-Y, Lewellen RT, Acosta-Leal R (2006) The continuing saga of rhizomania of sugar beets in the United States. Phytopathology 90: 4–15. 10.1094/PD-90-0004 30786468

[2] Rush CM (2003) Ecology and epidemiology of Benyviruses and plasmodiophorid vectors. Ann Rev Phytopathol 41: 567–592. 14527334 10.1146/annurev.phyto.41.052002.095705

[3] Casarini B (1999) Le avversità: loro natura, prevenzione e lotta. *In* La barbabietola negli ambienti mediterranei. Casarini B, Biancardi E, Ranalli P (eds.) Bologna, Italy: Edagricole. pp 273–421.

[4] Tamada T, Shirako Y, Abe H, Saito M, Kigushi T, et al (1989) Production and pathogenicity of isolates of *beet necrotic yellow vein virus* with different numbers of RNA components. J Gen Virol 70: 3399–3409.

[5] Kiguchi T, Saito M, Tamada T (1996) Nucleotide sequence analysis of RNA-5 of five isolates of *beet necrotic yellow vein virus* and the identity of a deletion mutant. J Gen Virol 77 4: 575–580. 8627244 10.1099/0022-1317-77-4-575

[6] Koenig R, Haeberle AM, Commandeur U (1997) Detection and characterization of a distinct type of *beet necrotic yellow vein virus* RNA 5 in a sugar beet growing area in Europe. Arch Virol 142: 1499–1504. 9267459

[7] Tamada T (1999) Benyviruses. In: Webster RG, Granoff A (eds) Encyclopedia of Virology (2^nd^ edn). London, UK: Academic Press. pp 154–160.

[8] Koenig R, Luddecke P, Haeberle AM (1995) Detection of *beet necrotic yellow vein virus* strains, variants and mixed infections by examining single-strand conformation polymorphisms of immunocapture RT-PCR products. J Gen Virol 76: 2051–2055. 7636486 10.1099/0022-1317-76-8-2051

[9] Koenig R, Lennefors BL (2000) Molecular analysis of European A, B and P type sources of *beet necrotic yellow vein virus* and detection of the rare P type in Kazakhstan. Arch Virol 145: 1561–1570. 11003469 10.1007/s007050070076

[10] Tamada T, Kusume T, Uchino H, Kigushi T, Saito M (1996) Evidence that *beet necrotic yellow vein virus* RNA-5 is involved in symptom development of sugar beet roots. In: Sherwood JL, Rush CM (eds) Proceedings of the 3rd Symposium of the International Working Group on Plant Viruses with Fungal Vectors. Denver, American Society of Sugar Beet Technologists, Dundee, UK, pp 49–52

[11] Heijbroek W, Musters PMS, Schoone AHL (1999) Variation in pathogenicity and multiplication of *Beet necrotic yellow vein virus* in relation to the resistance of sugar beet cultivars. Eur J Plant Pathol 105: 397–405.

[12] Kruse M, Koenig R, Hoffmann A, Kaufmann A, Commandeur U, et al (1994) Restriction fragment length polymorphism analysis of reverse transcription-PCR products reveals the existence of two major strain groups of *beet necrotic yellow vein virus* . J Gen Virol 75: 1835–1842. 7913953 10.1099/0022-1317-75-8-1835

[13] Schirmer A, Link D, Cognat V, Moury B, Beuve M, et al (2005) Phylogenetic analysis of isolates of *Beet necrotic yellow vein virus* collected worldwide. J Gen Virol 86: 2897–2911. 16186246 10.1099/vir.0.81167-0

[14] Biancardi E, Lewellen RT, DeBiaggi M, Erichsen AW, Stevanato P (2002) The origin of rhizomania resistance in sugar beet. Euphytica 127: 383–397.

[15] Lewellen RT, Skoyen IO, Erichsen AW (1987) Breeding sugarbeet for resistance to rhizomania: Evaluation of host-plant reactions and selection for and inheritance of resistance, in: Proceedings of the 50^th^ Congress of the IIRB. Brussels, Belgium: International Institute for Beet Research. pp 139–156.

[16] Lewellen RT, Biancardi E (1990) Breeding and performance of rhizomania resistant sugar beet, in: Proceedings of the 53^rd^ Congress of the IIRB. Brussels, Belgium: International Institute for Beet Research. pp 79–87.

[17] Scholten OE, Jansen RC, Keizer LCP, De Bock TSM, Lange W (1996) Major genes for resistance to beet necrotic yellow vein virus (BNYVV) in *Beta vulgaris* . Euphytica 91: 331–339.

[18] Mannerlöf M, Lennefors B-L, Tenning P (1996) Reduced titer of BNYVV in transgenic sugar beets expressing the BNYVV coat protein. Euphytica 90: 293–296.

[19] Fecker LF, Koenig R, Obermeier C (1997) *Nicotiana benthamiana* plants expressing *beet necrotic yellow vein virus* (BNYVV) coat protein-specific scFv are partially protected against the establishment of the virus in the early stages of infection and its pathogenic effects in the late stages of infection. Arch Virol 142: 1857–1863. 9672643 10.1007/s007050050203

[20] Pavli OI, Kelaidi GI, Tampakaki AP, Skaracis GN (2011) The *hrpZ* gene of *Pseudomonas syringae* pv. *phaseolicola* enhances resistance to rhizomania disease in transgenic *Nicotiana benthamiana* and sugar beet. PLoS ONE 6 3: e17306. 21394206 10.1371/journal.pone.0017306 PMC3048869

[21] Andika IB, Kondo H, Tamada T (2005) Evidence that RNA silencing-mediated resistance to *Beet necrotic yellow vein virus* is less effective in roots than in leaves. MPMI 18: 194–204. 15782633 10.1094/MPMI-18-0194

[22] Lennefors B-L, Savenkov EI, Bensefelt J, Wremerth-Weich E, van Roggen P, et al (2006) dsRNA-mediated resistance to *Beet Necrotic Yellow Vein Virus* infections in sugar beet (*Beta vulgaris* L. ssp. *vulgaris*). Mol Breed 18: 313–325.

[23] Pavli OI, Panopoulos NJ, Goldbach R, Skaracis GN (2010) BNYVV-derived dsRNA confers resistance to rhizomania disease of sugar beet as evidenced by a novel transgenic hairy root approach. Transgenic Res 19: 915–922. 20127510 10.1007/s11248-010-9364-y PMC2935974

[24] Tamada T, Miyanishi M, Kondo H, Chiba S, Han CG (2002) Pathogenicity and molecular variability of *Beet necrotic yellow vein virus* isolates from Europe, Japan, China, and the United States. In: Proceedings of the 5th Symposium of the International Working Group on Plant Viruses with Fungal Vectors. Denver, American Society of Sugar Beet Technologists, Zurich, Switzerland. pp. 13–16.

[25] Chiba S, Kondo H, Miyanishi M, Andika IB, Han C, et al (2011) The Evolutionary History of *Beet necrotic yellow vein virus* Deduced from Genetic Variation, Geographical Origin and Spread, and the Breaking of Host Resistance. Mol Plant Microbe Interact 24 2: 207–218. 20977309 10.1094/MPMI-10-10-0241

[26] Chiba S, Miyanishi M, Andica IB, Kondo H, Tamada T (2008) Identification of amino acids of the beet necrotic yellow vein virus p25 protein required for induction of the resistance response in leaves of *Beta vulgaris* plants. J Gen Virol 89: 1314–1323. 18420811 10.1099/vir.0.83624-0

[27] Acosta-Leal R, Rush CM (2007) Mutations associated with resistance-breaking isolates of Beet necrotic yellow vein virus and their allelic discrimination using TaqMan technology. Phytopathology 97: 325–330. 18943652 10.1094/PHYTO-97-3-0325

[28] Acosta-Leal R, Bryan BK, Smith JT, Rush CM (2010) Breakdown of host resistance by independent evolutionary lineages of *Beet necrotic yellow vein virus* involves a parallel C/U mutation in its *p25* gene. Phytopathology 100: 127–133. 20055646 10.1094/PHYTO-100-2-0127

[29] Acosta-Leal R, Fawley MW, Rush CM (2008) Changes in the intraisolate genetic structure of *Beet necrotic yellow vein virus* populations associated with plant resistance breakdown. Virology 376: 60–68. 18423510 10.1016/j.virol.2008.03.008

[30] Koenig R, Loss S, Specht J, Varrelmann M, Luddecke P, et al (2009) A single U/C nucleotide substitution changing alanine to valine in the beet necrotic yellow vein virus p25 protein promotes increased virus accumulation in roots of mechanically inoculated, partially resistant sugar beet seedlings. J Gen Virol 90: 759–763. 19218223 10.1099/vir.0.007112-0

[31] Pavli OI, Prins M, Goldbach R, Skaracis GN (2011) Efficiency of *Rz1*-based rhizomania resistance and molecular studies on BNYVV isolates from sugar beet cultivation in Greece. Eur J Plant Pathol 130: 133–142.

[32] Grimmer MK, Trybush S, Hanley S, Francis SA, Karp A, et al (2007) An anchored linkage map for sugar beet based on AFLP, SNP and RAPD markers and QTL mapping of a new source of resistance to *Beet necrotic yellow vein virus.* . Theor Appl Genet 114: 1151–1160. 17333102 10.1007/s00122-007-0507-3

[33] Tampakaki AP, Panopoulos NJ (2000) Elicitation of hypersensitive cell death by extracellularly targeted HrpZ*_Psph_* produced in planta. Mol Plant Microbe Interact 13 12: 1366–1374. 11106029 10.1094/MPMI.2000.13.12.1366

[34] Horsch R, Fry J, Hoffman N, Eichholtz D, Rogers S, et al (1985) A simple and general method for transferring genes into plants. Science 227: 1229–1231. 17757866 10.1126/science.227.4691.1229

[35] Dong H-P, Yu H, Bao Z, Guo X, Peng J, et al (2005) The ABI2-dependent abscisic acid signaling controls HrpN-induced drought tolerance in *Arabidopsis* . Planta 221: 313–327. 15599761 10.1007/s00425-004-1444-x

[36] Ren H, Song T, Wu T, Sun L, Liu Y, et al (2006) Effects of a biocontrol bacterium on growth and defense of transgenic rice plants expressing a bacterial type-III effector. Ann Microbiol 56: 281–287.

[37] Chen L, Zhang SJ, Zhang SS, Qu S, Ren X, et al (2008) A fragment of the *Xanthomonas oryzae* pv. *oryzicola* harpin HpaG_Xooc_ reduces disease and increases yield of rice in extensive grower plantings. Phytopathology 98: 792–802. 18943255 10.1094/PHYTO-98-7-0792

[38] Huo R, Wang Y, Ma L-L, Qiao J-Q, Shao M, et al (2010) Assessment of inheritance pattern and agronomic performance of transgenic rapeseed having harpin_Xooc_-encoding *hrf2* gene. Transg Res DOI 10.1007/s11248-010-9365-x. 10.1007/s11248-010-9365-x 20107894

[39] Oh CS, Beer SV (2007) AtHIPM, an Ortholog of the apple HrpN-Interacting Protein, is a negative regulator of plant growth and mediates the growth-enhancing effect of HrpN in *Arabidopsis* . Plant Physiol 145: 426–436. 17704235 10.1104/pp.107.103432 PMC2048737

[40] Shao M, Wang J, Dean RA, Lin Y, Gao X, et al (2008) Expression of a harpin-encoding gene in rice confers durable nonspecific resistance to *Magnaporthe grisea* . Plant Biotechnol J 6: 73–81. 18005094 10.1111/j.1467-7652.2007.00304.x

[41] Conrath U, Beckers GJM, Flors V, Garcia-Agustin P, Jakab G, et al (2006) Priming: Getting ready for battle. Mol Plant Microbe Interact 19: 1062–1071. 17022170 10.1094/MPMI-19-1062

